# Discovery of Heterotopic Bone-Inducing Activity in Hard Tissues and the TGF-β Superfamily

**DOI:** 10.3390/ijms19113586

**Published:** 2018-11-13

**Authors:** Takenobu Katagiri, Sho Tsukamoto, Yutaka Nakachi, Mai Kuratani

**Affiliations:** 1 Division of Pathophysiology, Research Center for Genomic Medicine, Saitama Medical University, Saitama 350-1241, Japan; stsukamo@saitama-med.ac.jp (S.T.); nakachiy@saitama-med.ac.jp (Y.N.); kuratani@saitama-med.ac.jp (M.K.); 2 Project of Clinical and Basic Research for FOP, Saitama Medical University, Saitama 350-1241, Japan; 3 Division of Pathophysiology, Research Center for Genomic Medicine, Saitama Medical University, 1397-1 Yamane, Hidaka-shi, Saitama 350-1241, Japan

**Keywords:** bone induction, demineralization, bone morphogenetic protein, signaling, fibrodysplasia ossificans progressiva

## Abstract

Bone is a unique organ because it can be experimentally induced in soft tissues by implanting a single growth factor, bone morphogenetic protein (BMP). Heterotopic bone-inducing activity was found in demineralized bone matrix in 1965. The characterization of this activity in bone enabled the purification and molecular cloning of BMPs and showed that they are members of the transforming growth factor-β (TGF-β) superfamily. Assay systems developed for this bone-inducing activity revealed the molecular mechanisms of the intracellular signaling of members of the superfamily, including BMPs. Moreover, they are being applied to elucidate molecular mechanisms and to develop novel therapeutics for a disease caused by an abnormality in BMP signaling.

## 1. Introduction

Bone is one of the mineralized hard organs in vertebrates. Bone consists of organic and inorganic components, such as type I collagen and hydroxyapatite crystals, respectively. Bone not only stores calcium and phosphate ions but also acts as a source of various blood cells by forming bone marrow in adulthood. Similar to other organs, functional bone is regenerated after damage, including fractures, through a cascade of bone formation similar to that seen during embryogenesis. The site of organ regeneration is usually limited to the location of the original organ. However, functional bone containing bone marrow can be induced experimentally in various soft tissues in vertebrates by a single growth factor. This capacity of heterotopic organ induction in vivo is unique to bone and is not observed in other organs, such as the skeletal muscle, heart, brain, liver, or kidney.

The unique activity of heterotopic bone induction was originally observed in a demineralized dead bone matrix by Urist in 1965 [1] and named “bone morphogenetic protein (BMP)” due to its biochemical characteristics. The purification and molecular cloning of BMP from bone revealed that the heterotopic bone-inducing BMP activity resulted from a mixture of several members of the transforming growth factor-β (TGF-β) superfamily [2]. No other growth factors, hormones, chemokines, and small chemical compounds induce heterotopic bone formation in soft tissues in vivo without activating the BMP signaling pathway. BMPs play critical roles in skeletal development during embryogenesis, tissue regeneration and pathological conditions in skeletal diseases [3,4]. In this review article, we focus on the discovery of heterotopic bone-inducing activity and the application of knowledge about BMPs to a genetic disease caused by an abnormality in BMP signaling.

## 2. A Heterotopic Bone-Inducing Activity in Bone and Teeth

Experimental “heterotopic bone induction” in vivo has been reported to be achieved by transplanting gall bladder and urinary bladder epithelial cells into soft tissues, such as the abdominal wall and muscle [5,6,7,8]. Newly formed bone and bone marrow containing red blood cells were observed at the transplant sites. There were multiple possibilities for this new bone formation; the epithelial cells might have transdifferentiated into osteoblasts in the host animals, or the donor epithelial cells might have induced a cascade of bone formation by the host cells.

In 1965, Urist [1] reported heterotopic bone induction using bone matrix. He prepared a demineralized bone matrix by treating long bones with 0.6 M hydrochloride (HCl) to remove minerals and transplanted it into a skeletal muscle pouch in the host animals. Several weeks later, the transplanted bone matrix was detected in an X-ray analysis as remineralized matrix. Moreover, the remineralized matrix contained bone-forming osteoblasts and osteocytes embedded in the newly formed bone matrix, chondrocytes in cartilaginous matrices, and bone marrow, even though the donor cells had been killed by treatment with 0.6 M HCl for several days. This finding suggested that the bone matrix contained a new bone-inducing activity resulting from “autoinduction” [1], which may induce a whole cascade of endochondral bone formation in skeletal muscle. Demineralized dentin, but not enamel, also showed such heterotopic bone-inducing activity in vivo [9,10]. Both human and mouse osteosarcomas were found to have this bone-inducing activity [11,12,13,14].

Bone and dentin are mineralized through at least two steps. First, osteoblasts and odontoblasts secrete organic components, including type I collagen and other non-collagenous proteins. Then, the organic matrix is mineralized by hydroxyapatite crystals consisting of calcium and phosphate ions. Several components stored in the mineralized organs, such as osteocalcin, are bound directly to hydroxyapatite crystals and thus are released from the hard tissues by demineralization [15]. In contrast, the bone-inducing activity of both bone and dentin was still found in the demineralized matrix, suggesting that this activity was closely associated with organic components rather than with hydroxyapatite crystals. However, the mineralization of bone matrix had been reported to be important for bone-inducing activity in vivo. Bone-inducing activity was significantly lower in vitamin D-deficient rachitic rats than in control rats [16]. A high-calcium diet in vitamin D-deficient rats restored the bone-inducing activity in the bone matrix [17]. Moreover, a low-calcium diet reduced the bone-inducing activity in rats even in the presence of vitamin D, suggesting that mineralization of the bone matrix rather than vitamin D itself regulates the amount of this activity in bone [17]. This finding suggests that the material responsible for the bone-inducing activity is accumulated during the mineralization process rather than by the secretion of organic components by osteoblasts. It is possible that osteoblasts secrete the materials responsible for the bone-inducing activity during the mineralization process. Alternatively, the materials responsible for the bone-inducing activity are secreted by other organs and accumulate through circulation in the bone matrix, as several bioactive BMPs were found in circulation in vertebrates [18,19,20,21,22,23].

## 3. Biochemical Characteristics of the Bone-Inducing Activity in Bone Matrix

An important biochemical characteristic of the bone-inducing activity in demineralized bone matrix was elucidated by digestion with two types of enzymes, α-amylase and trypsin, which mainly target carbohydrate chains and proteins, respectively [24]. Digestion with α-amylase released a large amount of hexosamine from the demineralized bone matrix but did not change the volume of new bone induced by the bone matrix. In contrast, digestion with trypsin reduced the bone volume to less than 10% of that in the controls, suggesting that bone-inducing activity belongs to a proteinous component in the bone matrix [24]. Based on this finding, the source of the bone-inducing activity in demineralized bone matrix was named “bone morphogenetic protein (BMP)” [24]. The bone-inducing activity was inhibited by treatment with sulfhydryl reducing agents, such as dithiothreitol and 2-mercaptoethanol, suggesting that disulfide bond(s) are essential for the activity [25]. This suggestion is supported by a biochemical characteristic of the TGF-β superfamily: They act as dimerized proteins through a disulfide bond [4].

The bone-inducing activity in demineralized bone matrix was found to be diffusible through cellulose acetate membranes in vivo [26,27]. In these experiments, demineralized bone matrix was placed inside a diffusion chamber, which prevented cellular invasion but allowed the diffusion of small components through micropore membranes. Heterotopic bone was formed on the outer layer of the membranes, indicating that the active molecule in the demineralized bone matrix was released from the matrix and induced the differentiation of target cells outside the chamber even through five multilayered membranes. These data also suggest that the material responsible for the bone-inducing activity can be extracted from demineralized bone matrix.

The material responsible for the bone-inducing activity in bone was solubilized by treatment with guanidine HCl [28]. Treatment with guanidine HCl or high concentrations of urea with 1 M NaCl slightly reduced the amount of hydroxyproline in the matrix but significantly reduced the bone-inducing activity, suggesting that the material responsible for the activity was inactivated or released from collagenous matrices [29,30]. The addition of 1 M NaCl to 8 M urea efficiently abolished the bone-inducing activity in the matrix [30]. The solubilized extracts of demineralized bone matrix or osteosarcoma obtained by using guanidine HCl exhibited the bone-inducing activity, although the extracted residues lost this activity [13,28,30,31]. Reconstruction of the solubilized extracts with the residue efficiently induced bone formation in soft tissues, although the residue alone did not induce bone formation [30]. This assay system in vivo, involving reconstitution with the extracted residues, was widely used to evaluate the heterotopic bone-inducing activity of the fractions purified from bone and the recombinant proteins produced by molecular engineering. The shape of the new bone induced is dependent on the shape of the transplants. The organic residues act as carriers for suppressing diffusion and maintaining effective local concentrations of the active molecules. Moreover, they may facilitate the invasion of progenitor cells such as osteoblasts and/or chondrocytes at the local sites in vivo. Not only collagen but also synthetic resorbable polymers were developed as carriers for bone induction by BMPs in vivo.

The material responsible for bone-inducing activity in bone was found to bind to heparin, and therefore, heparin–Sepharose affinity chromatography was used for purification of the material responsible for the activity from the extracts [32,33]. The bone-inducing activity in demineralized bone matrix was suppressed by treatment with heparin [34,35], but heparin enhanced heterotopic bone formation by BMP-2 due to the efficient supply of the ligand to the receptors, provided by protecting ligands from accumulation in the extracellular matrices [36,37,38]. Other types of sulfated polysaccharides also enhanced BMP activity in vitro [37], suggesting that negatively charged sulfated polysaccharides in the extracellular matrices, such as proteoglycans, are physiological binding sites of BMPs that regulate this activity. The components of the BMP complex, such as proteoglycans and collagens, and the mechanisms of regulation of natural BMP activity by furin-like enzymes under physiological conditions remain to be elucidated in future research.

## 4. Purification and Identification of Members of the TGF-β Superfamily from Demineralized Bone Matrix

Cartilage is formed in skeletal muscle by demineralized bone matrix, not only in vivo but also in vitro systems. In cultures of small pieces of thigh muscle of newborn rats placed on a demineralized bone matrix, fibroblastic mesenchymal cells grew from the specimens and differentiated into chondrocytes to form hyaline cartilage on the bone matrix [39,40,41]. Artificial crevices made in the demineralized bone matrix with a scalpel enhanced cartilage formation by skeletal muscle cells in vitro [42]. Lower-oxygen conditions showed more hypertrophic chondrocytes in the organ cultures [42]. The chondrogenesis of skeletal muscle cells in vitro was also observed using extracts of demineralized bone matrix. Thus, this assay system was used to purify the material responsible for “cartilage-inducing activity” in the bone matrix [43,44,45]. TGF-β1 and TGF-β2 were found to be cartilage-inducing factor-A and cartilage-inducing factor-B, respectively [44,45]. TGF-β itself did not induce heterotopic bone formation in vivo or the osteoblastic differentiation of muscle cells in vitro [32,46]. However, some non-osteogenic members of the TGF-β superfamily synergistically enhanced the heterotopic bone-inducing activity of osteogenic BMP in vivo [47,48,49].

**Figure 1:**
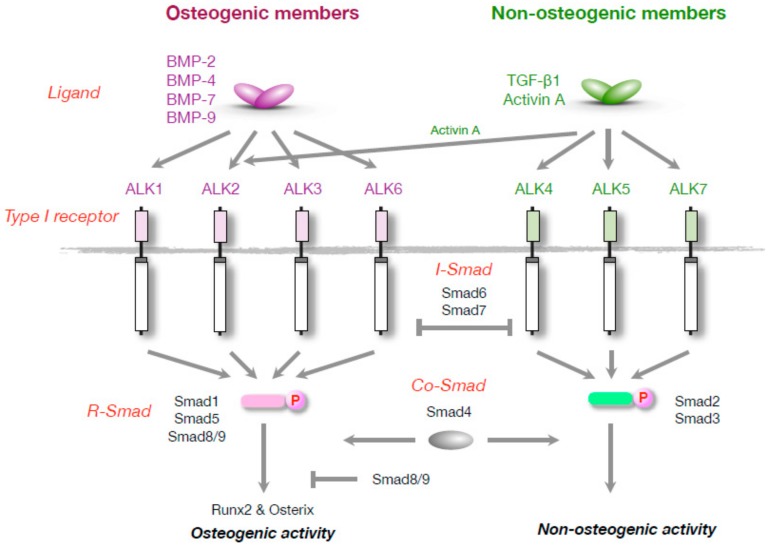
Schematic representation of signal transduction by osteogenic and non-osteogenic members of the transforming growth factor-β (TGF-β) superfamily.

The highly purified fraction of the material responsible for bone-inducing activity from demineralized bone matrix showed a molecular mass of approximately 30 kDa in a non-reduced condition [33,50,51]. Wozney and colleagues [2] successfully cloned novel cDNAs for BMP-1, BMP-2/2a, BMP-3 and BMP-4/2b using the amino acid sequences of the tryptic peptides obtained from the highly purified active fractions. Except for BMP-1, which is a metalloproteinase, the predicted sequences of the BMPs identified the BMPs as novel members of the TGF-β superfamily [2]. Other members of the TGF-β superfamily, such as BMP-3/osteogenin, BMP-5, BMP-6 and BMP-7, were also cloned using a similar strategy [2,50,51,52,53]. The heterotopic bone-inducing activity of each BMP, including BMP-2, BMP-4 and BMP-7, was evaluated using recombinant proteins expressed in vitro in the in vivo reconstitution assay (Figure 1) [2,51,52,53]. Some other members of the TGF-β superfamily, including Drosophila decapentaplegic (Dpp) and 60 A, even though they are derived from organisms that do not have mineralized bone, have also been shown to have heterotopic bone-inducing activity in vivo [54]. These findings revealed that the bone-inducing activity observed in demineralized bone matrix resulted not from a single molecule but from a mixture of several members of the TGF-β superfamily. In addition, the intracellular signaling of BMPs is highly conserved from Drosophila to humans, and phenotypes such as bone induction and morphogenesis are dependent on the types of target cells but not on ligands. The latter possibility was supported by the finding that human BMP-4 rescued dorsal–ventral patterning in a Dpp mutant in Drosophila [55].

## 5. Intracellular Signaling of the TGF-β Superfamily to Induce Heterotopic Bone Formation

The molecular mechanisms of intracellular signaling involved in heterotopic bone induction by BMPs were examined using various cell lines in vitro. Murine C2C12 myoblasts, which are derived from regenerating thigh muscle, are one of the most widely used cell lines in research on myogenesis [56]. C2C12 cells express MyoD and proliferate as immature mononuclear myoblasts, differentiate into mature myocytes, and form multinucleated myotubes expressing skeletal muscle-specific contracting proteins in vitro. BMP-2 inhibited the formation of myotubes and the expression of skeletal muscle-related proteins [46]. Moreover, the cells treated with BMP-2 expressed osteoblastic markers, such as high alkaline phosphatase activity, cAMP accumulation in response to parathyroid hormone, and the secretion of osteocalcin [46]. TGF-β1 also inhibited the myogenesis of C2C12 cells similarly to BMP-2, but TGF-β1 did not induce such osteoblastic markers, suggesting that this in vitro model using C2C12 cells reflects the heterotopic bone-inducing activity of BMPs [46]. Using an adenoviral expression system, the biological activity of BMP-2 through BMP-15 was examined in C2C12 cells. BMP-2, BMP-6 and BMP-9 were found to induce high levels of ALP activity in the cells [57]. In contrast to primary skeletal muscle cells, chondrogenesis (evaluated by round-shaped morphology and Alcian blue staining) was not detected in the C2C12 cultures treated with BMP-2 (unpublished observation).

Members of the TGF-β superfamily bind to transmembrane serine-threonine kinase receptors on the surface of target cells. These receptors are classified into type I and type II by the presence and absence, respectively, of a glycine- and serine-rich domain (GS domain) in juxtamembrane intracellular domains. Among 7 type I receptors (ALK1 through ALK7), ALK1, ALK2/ACVR1, ALK3/BMPR-IA and ALK6/BMPR-IB bind to BMPs. Among 4 type II receptors, BMPR-II, ActR-IIA and ActR-IIB bind to BMPs, although they also bind to other members of the TGF-β superfamily. Type I receptors are phosphorylated at the GS domain by type II receptor kinases as substrates in response to ligand stimulation. BMPs directly bind type I receptors, although TGF-β and activin require type II receptors for binding to type I receptors [58]. The substitution of a specific residue in the GS domain of type I receptors constitutively activates intracellular signaling without the addition of exogenous ligands or the activation of type II receptors [59]. Radio-labeled BMPs bind to BMPR-II, ActR-IIA, ActR-IIB, ALK2 and ALK3 in C2C12 cells [60]. The overexpression of constitutively active forms of ALK1, ALK2/ACVR1, ALK3/BMPR-IA and ALK6/BMPR-IB, induces ALP activity in C2C12 cells, suggesting that they function as type I receptors for osteogenic BMPs [61,62].

Type I receptor kinases phosphorylate downstream effectors in cytoplasm (Figure 1). Smad proteins (Smad1, Smad5 and Smad8/9) are critical substrates in signaling for the osteogenic activity of BMPs [63,64]. These Smad proteins have a conserved serine–valine–serine (SVS) motif at the carboxy terminus, and both serine residues are phosphorylated by type I receptors in response to ligand binding. A mutant of Smad1 in which the serine residues in the SVS motif are replaced by glutamic acids (DVD), was recognized by an antiphosphorylated Smad1/5/8 polyclonal antibody, even though the glutamic residues were not phosphorylatable by type I receptor kinases, suggesting that the structure of the C-terminus of the DVD form of Smad1 is similar to that of phosphorylated Smad1 [65]. Moreover, transient overexpression of the DVD forms of Smad1, Smad5 and Smad8/9 in C2C12 cells induced BMP signaling and osteoblastic differentiation, suggesting that the intracellular signaling pathway through Smad1, Smad5 and Smad8/9 is sufficient for the osteogenic activity of BMPs [65,66]. The transcriptional activity of Smad8/9 was significantly lower than that of the transcriptional activity of Smad1 and Smad5 due to a unique sequence in the linker region [66]. Smad8/9 acts as not only a positive effector but also a dominant negative effector in BMP signaling [66]. Smad8/9 weakly activated a BMP-specific luciferase reporter but suppressed the reporter activity induced by a constitutively active form of Smad1, Smad1(DVD) [66]. Smad6 and Smad7 inhibited BMP signaling by blocking the kinase activity of type I receptors but did not block Smad1(DVD) [66]. In contrast, Smad8/9 suppressed BMP signaling as a dominant-negative Smad against Smad1 and Smad5 by binding to Smad4 and a target DNA element [66]. The Smad proteins activated by phosphorylation at the SVS motif by BMP type I receptors regulate the transcription of target genes through binding to specific DNA sequences. The expression of the *Id1* gene was increased within 1 h after BMP-2 stimulation in C2C12 cells [46]. A GC-rich BMP response element was identified in the 5′ enhancer region in the human and mouse *Id1* gene, which was recognized by Smad1 and Smad4—a coactivator of the phosphorylated Smad proteins [64,65,66,67,68,69]. Similar GC-rich BMP-responsive elements were found in the *Id2*, *Id3* and *BIT-1* genes [70]. Luciferase reporters driven by BMP-responsive elements are used to monitor BMP signaling in various types of cells in vitro and in vivo [67,68,69,70,71,72].

The differentiation of osteoblasts is regulated by some specific transcription factors. Osterix/Sp7 has been identified as a novel transcription factor containing zinc-finger motifs from C2C12 cells stimulated by BMP-2 [73]. Osterix-knockout mice are deficient in mineralized bone tissue due to a lack of bone-forming osteoblasts. Interestingly, these mice still express another transcription factor essential for osteoblast differentiation, Runx2/Cbfa1, indicating that Runx2/Cbfa1 is upstream of Osterix in the osteoblast differentiation pathway.

## 6. BMP Signaling in a Genetic Disorder That Involves Heterotopic Bone Formation in Skeletal Muscle

Heterotopic bone formation through endochondral ossification in soft tissues, such as skeletal muscle, tendons and ligaments, occurs in patients with a rare genetic disease, fibrodysplasia ossificans progressiva (FOP) (OMIM #135100). This phenotype seems to be similar to bone induction by BMPs in skeletal muscle, but the gene responsible for this disease has not been identified. In 2006, a recurrent substitution mutation in the GS domain of ALK2/ACVR1 was found in patients with typical FOP [74]. Overexpression of this mutant ALK2/ACVR1 in vitro activated a BMP signaling pathway, including the phosphorylation of Smad1 and Smad5, activation of *Id1* transcription and induction of the ALP activity of C2C12 cells [75,76,77]. These results suggest that heterotopic bone formation in patients with FOP is caused by BMP signaling through ALK2/ACVR1. FOP is the first example of a naturally occurring gain-of-function mutation among type I receptors of the TGF-β superfamily.

However, the activity of the mutant ALK2/ACVR1 associated with FOP was much weaker than that of a constitutively active form developed by molecular engineering [78]. Moreover, the mutants of ALK2/ACVR1 were further enhanced by the co-expression of a BMP type II receptor in a kinase activity-dependent manner, suggesting that they still require the formation of a tertiary complex with the ligand and type II receptor for full activation [78,79]. This possibility was supported by the identification of activin A as a trigger of heterotopic ossification in FOP model mice [80,81]. In association with typical FOP, the mutant but not the wild-type ALK2/ACVR1 activates BMP signaling through Smad1 and Smad5 in response to activin A binding [80,82]. This result seemed to be a “neofunction” of activin A in FOP [82]. In patients with FOP, heterotopic ossification is induced by trauma, suggesting that inflammatory responses activate the activin A-mutant ALK2/ACVR1 axis in vivo. The depletion of mast cells and/or macrophages reduced the volume of heterotopic ossification in the FOP model mice [83]. The chondrocytes and osteoblasts that appeared during heterotopic bone formation induced by BMP signaling, including a FOP model, were not derived from myogenic cells but from interstitial mesenchymal cells in skeletal muscle and tendon-derived cells in vivo [81,84,85]. These findings on FOP clearly indicated that intracellular signaling through Smad1 and Smad5 by activation of ALK2/ACVR1 is important for heterotopic bone formation in humans. Various inhibitors of intracellular signaling through ALK2/ACVR1 at multiple steps have been developed to establish effective therapeutics for FOP [86,87,88]. Anti-activin A neutralizing antibody, a Smad inhibitor (retinoic acid receptor γ agonist), and a chondrocyte differentiation inhibitor (rapamycin) have been examined in clinical trials of patients with FOP [88]. Kinase inhibitors specific for ALK2/ACVR1 and an anti-ALK2/ACVR1 blocking antibody are also under development for FOP. In vivo studies using these inhibitors may reveal novel functions of ALK2/ACVR1 among the type I receptors of the TGF-β superfamily.

## 7. Conclusions

The molecular mechanisms of heterotopic bone induction by demineralized bone matrix in skeletal muscle have been elucidated over the last five decades. The elucidation of the biochemical characteristics of the bone-inducing activity in demineralized bone matrix has enabled the purification and molecular cloning of BMPs. The molecular cloning of BMPs revealed that they are the largest subfamily in the TGF-β superfamily. The assay systems developed in vivo and in vitro for this heterotopic bone-inducing activity identified functional receptors and intracellular effectors of BMPs. These assay systems and knowledge of BMPs are being applied to elucidate molecular mechanisms and to develop novel therapeutics for diseases caused by abnormalities in BMP signaling, such as FOP. Inhibitors and/or potentiators of BMP activity that act in the extracellular or intracellular spaces will be applicable for other skeletal diseases, such as fracture healing, nonunion and multiple hereditary exostoses. To treat these diseases, classic basic questions about BMPs remain to be clarified, for example; when are BMPs stored in the bone matrix? How are BMPs activated in native bone matrix to show bone-inducing activity? What types of cells are targets of physiological and pathological bone formation induced by BMPs?
